# Ponatinib Activates an Inflammatory Response in Endothelial Cells via ERK5 SUMOylation

**DOI:** 10.3389/fcvm.2018.00125

**Published:** 2018-09-06

**Authors:** Jesus Paez-Mayorga, Andrew L. Chen, Sivareddy Kotla, Yunting Tao, Rei J. Abe, Emma D. He, Brian P. Danysh, Marie-Claude C. Hofmann, Nhat-Tu Le

**Affiliations:** ^1^Department of Cardiovascular Sciences, Center of Cardiovascular Regeneration Houston, Methodist Research Institute, Methodist Hospital, Houston, TX, United States; ^2^Tecnologico de Monterrey, Escuela de Medicina y Ciencias de la Salud, Monterrey, Mexico; ^3^Department of Cardiology, The University of Texas MD Anderson Cancer Center, Houston, TX, United States; ^4^Department of Endocrine Neoplasia and Hormonal Disorders, University of Texas MD Anderson Cancer Center, Houston, TX, United States

**Keywords:** ponatinib, vascular adverse events, ERK5 SUMOylation, EC inflammation, tyrosine kinase inhibtor (TKI)

## Abstract

Ponatinib is a multi-targeted third generation tyrosine kinase inhibitor (TKI) used in the treatment of chronic myeloid leukemia (CML) patients harboring the Abelson (Abl)-breakpoint cluster region (Bcr) T315I mutation. In spite of having superb clinical efficacy, ponatinib triggers severe vascular adverse events (VAEs) that significantly limit its therapeutic potential. On vascular endothelial cells (ECs), ponatinib promotes EC dysfunction and apoptosis, and inhibits angiogenesis. Furthermore, ponatinib-mediated anti-angiogenic effect has been suggested to play a partial role in systemic and pulmonary hypertension via inhibition of vascular endothelial growth factor receptor 2 (VEGFR2). Even though ponatinib-associated VAEs are well documented, their etiology remains largely unknown, making it difficult to efficiently counteract treatment-related adversities. Therefore, a better understanding of the mechanisms by which ponatinib mediates VAEs is critical. In cultured human aortic ECs (HAECs) treated with ponatinib, we found an increase in nuclear factor NF-kB/p65 phosphorylation and NF-kB activity, inflammatory gene expression, cell permeability, and cell apoptosis. Mechanistically, ponatinib abolished extracellular signal-regulated kinase 5 (ERK5) transcriptional activity even under activation by its upstream kinase mitogen-activated protein kinase kinase 5α (CA-MEK5α). Ponatinib also diminished expression of ERK5 responsive genes such as Krüppel-like Factor 2/4 (*klf2/4*) and *eNOS*. Because ERK5 SUMOylation counteracts its transcriptional activity, we examined the effect of ponatinib on ERK5 SUMOylation, and found that ERK5 SUMOylation is increased by ponatinib. We also found that ponatibib-mediated increased inflammatory gene expression and decreased anti-inflammatory gene expression were reversed when ERK5 SUMOylation was inhibited endogenously or exogenously. Overall, we propose a novel mechanism by which ponatinib up-regulates endothelial ERK5 SUMOylation and shifts ECs to an inflammatory phenotype, disrupting vascular homeostasis.

## Introduction

CML and Ph^+^ALL involve the reciprocal translocation of the Abl oncogene on chromosome 9 and the Bcr on chromosome 22 (1–3). The resulting chromosomal fusion produces a constitutively active Bcr-Abl tyrosine kinase (4) that promotes dysregulated proliferation and survival signaling, leading to leukemogenesis (1, 5). Therefore, Bcr-Abl kinase is the primary therapeutic target for CML patients. Newly diagnosed CML patients commonly receive imatinib (first generation TKI), a small molecule that binds the ATP pocket on the Bcr-Abl tyrosine kinase, as a first line of treatment (6, 7). Because CML patients often develop Bcr-Abl point mutations conferring resistance to imatinib (8), dasatinib, and nilotinib (second generation TKIs) were generated (9). T315I is a specific point mutation present in ~15% of relapsed CML patients (10) that causes therapeutic resistance to all currently approved first and second generation TKIs (11–13). Ponatinib (a third generation TKI) was specifically designed to circumvent the sterical hindrance warranted by the T315I mutation (14–17). Since it is the only drug effective against this mutation, ponatinib has become the treatment of choice for CML patients harboring T315I Bcr-Abl (18–23).

In spite of having superb clinical efficacy, ponatinib treatment comes with an array of adverse side effects attributable to the broad-spectrum inhibition of multiple kinase families in addition to Bcr-Abl (3). Common secondary effects of ponatinib treatment are xerostomia, abdominal pain, and cytopenia. Specifically in the cardiovascular system, ponatinib treatment induces substantial arterial and venous VAEs (24) including peripheral arterial occlusive disease (25), ischemic heart disease (26), cerebrovascular accident, venous thrombo-embolism (27), pulmonary hypertention (28), platelet dysfunction, and hyperglycemia (26–30). In a prospective analysis of 19 patients who received ponatinib therapy, 42% developed arterial cardiovascular events after 8.5 months. A phase I trial showed a significant percentage of vascular occlusive events (24, 31) and a phase II trial (PACE) demonstrated a strong correlation between ponatinib administration and serious arterial thrombotic events (10, 32). A randomized, opened-label phase III trial (EPIC) designed to compare efficacy between ponatinib and imatinib as first line treatments in newly diagnosed CML patients was terminated early due to serious VAEs observed in the ponatinib treated group (33). Ponatinib-associated VAEs are a serious clinical challenge in CML patients subjected to this therapeutic regime (34). A broad comparative profiling analysis of ponatinib and other TKIs showed that ponatinib inhibits VEGFRs with greater potency (26), through which it reduces viability, function, migration, and tube formation in ECs, thus causing vascular toxicity (35). Ponatinib-associated VEGFR2 inhibition has also been implicated in hypertension (26).

Even though ponatinib-mediated VAEs have been documented (36, 37) the exact molecular mechanism by which this drug induces VAEs remains obscure. Interestingly, despite promoting arterial thrombotic events (26, 38), ponatinib inhibits platelet activation, aggregation, spreading, and granule secretion (39). These observations suggest that ponatinib-associated thrombotic events are not due to the activation of platelets (40), but rather of other cell types. Because ponatinib treatment increases EC dysfunction and apoptosis (35), both of which are associated with a higher rate of VAEs (41), it is plausible that ponatinib-associated VAEs are related to EC inflammation, dysfunction and apoptosis.

In ECs, ERK5 plays an important role in maintaining vascular homeostasis (42). Similar to ERK1/2, ERK5 has the activation loop (T-x-Y sequence) on its dual phosphorylation sites (T218/Y220) (43) as well as a kinase domain on the NH2-terminus. Uniquely, ERK5 contains two transcriptional activation domains on the COOH-terminus (44–46), giving it a different function and regulatory mechanism from ERK1/2 (45, 47, 48). ERK5 is activated by a wide range of stimuli, among which is laminar flow with anti-inflammatory and anti-atherogenic properties (43). In its inactive form, the intra-molecular interaction between NH2-terminus and COOH-terminus of ERK5 inhibits ERK5 transcriptional activity (47). The activation of ERK5 by upstream regulators such as MEK5α and laminar flow (49, 50), disrupts this intra-molecular interaction and triggers T218/Y220 phosphorylation and subsequent transcriptional activation, conferring anti-inflammatory, anti-apoptotic, and anti-atherogenic properties (51–59). Under pro-inflammatory conditions, such as reactive oxygen species and disturbed flow, ERK5 is SUMOylated at K6/22 (60) and phosphorylated at S496 (52). These two post-translational modifications inhibit ERK5 transcriptional activity, resulting in EC inflammation and apoptosis (52, 61).

In the current study, we tested the hypothesis that ponatinib triggers an endothelial inflammatory response by promoting ERK5 SUMOylation.

## Methods

Antibodies used in this study are listed in Table 1.

**Table 1:** List of antibodies.

**Antibody**	**Vendor**	**Cat #**
Tubulin	Sigma	T9026
ERK5	Cell Signaling	3372
SUMO2/3	Abgent	Ap1224a
pERK5-S496	Abnova	PAB15918
pERK5-T218/Y220	Cell Signaling	3371
SENP2	Novus	NBP1-31217
VCAM1	Cell Signaling	13662
p-p65 NFkB	Cell Signaling	3033
p-65 NFkB	Cell Signaling	8242
β-Actin	Novus	NB600-532
p-p90RSK-S380	Cell Signaling	9341
RSK	R&D Systems	MAB2056

### Ponatinib preparation and treatment

Ponatinib was obtained from ARIAD pharmaceuticals. Following the manufacturer's instructions, ponatinib was dissolved in citrate buffer 25 mM pH 2.75 [2.5 mM sodium citrate (CAS no. 6132-04-3); 25 mM citric acid (CAS no. 77-92-9)]. Confluent, quiescent Human Umbilical Vein ECs (HUVECs) or Human Aortic ECs (HAECs) were treated with ponatinib or vehicle and incubated for the indicated times at 37°C.

### Generation of plasmids and adenoviruses

Constitutively active form of MEK5α (CA-MEK5α) plasmid, adenoviruses expressing ERK5 wild type (Ad-ERK5-WT), ERK5 non-SUMOylatable mutant (Ad-ERK5-K6/22R), ERK5 phosphorylation resistant mutant (Ad-ERK5-S496A), and Sentrin/SUMO-specific protease 2 (SENP2) were generated previously (33, 41, 44, 48–50). Where indicated, an adenovirus containing β-galactosidase (Ad-LacZ) was used as a control (33, 41, 44, 48–50).

### Cell culture

HUVECs were purchased from Lifeline cell technology (C-12200, cat. no. 10171-906). HAECs were a kind gift from Dr. Lusis (UCLA, David Geffen School of Medicine). HUVECs and HAECs were cultured in Petri dishes or flasks coated with 0.2% gelatin type A (cat. no. 901771; MP Biomedicals, Santa Ana, CA, USA), in Endothelial Cell Medium (ECM, Cat.no. 1001, ScienCell, Carlsbard, CA. USA) containing 465 mL of basal medium, 25 mL of fetal bovine serum (FBS, Cat. no. 0025, ScienCell, Carlsbard, CA, USA), 5 mL of Endothelial Cell Growth Supplement (ECGS, Cat. no. 1052, ScienCell, Carlsbard, CA, USA) and 5 mL of penicillin/streptomycin solution (P/S, Cat. no. 0503, ScienCell, Carlsbard, CA, USA). Only HUVECs with less than 6 passages and HAECs with <15 passages were used in this study.

### NF-κB activity assay

NF-κB activity was measured using a luciferase assay with a reporter gene containing five NF-κB-binding sites as an enhancer [pLuc-MCS with five repeated NF-κB–binding sites (TGGGGACTTTCCGC); Stratagene, La Jolla, CA, USA]. A transfection mixture was made using GIBCO Opti-MEM Reduced Serum Medium (cat. no. 31985070**;** Thermo Fisher Scientific, Waltham, MA, USA) to which DEAE-DEXTRAN (final concentration, 0.375 μg/μl, cat. no. D9885; Sigma, St. Louis, MO, USA), a reporter vector, and a pRL-CMV vector (Promega, Madison, WI, USA) were added, and the mixture was incubated for 10 min at 37°C. pRL-CMV was used as an internal control for Renilla luciferase activity. Next, culture medium was removed, cells were washed with PBS and the transfection mixture was added. After 90 min of incubation, cold Opti-MEM Reduced Serum Medium containing 5% dimethyl sulfoxide was added to the cells, and the mixture was incubated for an additional 5 min. Cells were then washed once with PBS and cultured in a normal ECM culture medium. At the completion of experiments, cells were harvested in a passive lysis buffer (cat. no. E1960; Promega, Madison, WI, USA), and the NF-κB activity was determined by using a GloMax 20/20 Luminometer (Promega, Madison, WI, USA) to measure luciferase activity in resulting cell lysates (dual-luciferase reporter assay system, cat. no. E1960; Promega, Madison, WI, USA), as we have described previously (41, 50). Relative NF-κB activity was calculated by normalizing firefly luciferase activity to Renilla luciferase activity (firefly: renilla luciferase activity ratio).

### qRT-PCR

At the end of experiments, ECs were washed three times with PBS, and lysed in RLT Plus RNeasy lysis buffer (cat. no. 74136; QIAGEN, Germantown, MD, USA). The resulting cell lysates were loaded onto a QIAshredder column (cat. no. 79656; QIAGEN, Germantown, MD, USA), and spun down to collect the eluted lysates. Total RNA was then isolated from this lysate using an RNeasy Plus Mini Kit (cat. no. 74136; QIAGEN, Germantown, MD, USA) following the manufacturer's instructions. cDNA reverse transcription was performed with a 50 μl reaction mixture containing 1 μg of purified RNA, 5 μl of 10X buffer, 11 μl of MgCl_2_, 10 μl of dNTPs, 2.5 μl of a random hexamer, 1.25 μl of oligo-dT, 1 μl of RNase inhibitor, and 0.75 μl of a reverse transcriptase enzyme using TaqMan Reverse Transcription Reagents (cat. no. N808-0234; made for Applied Biosystems by Roche Molecular Diagnostics, Pleasanton, CA, USA). First-strand cDNA was reverse-transcribed from total RNA by incubating reaction mixtures at 25°C for 10 min followed by 37°C for 60 min, 42°C for 60 min, and 95°C for 5 min before soaking at 4°C in a PCR cycler. Target cDNA levels were quantified using a CFX Connect Real-Time System (Bio-Rad, Hercules, CA, USA). Each reaction mixture (10 μl) contained cDNA synthesized from 20 ng of total RNA, 5 μl of iQ SYBR Green Supermix (cat. no. 1708882; Bio-Rad, Hercules, CA, USA), and 0.5 μmol/l each forward and reverse primer (see Table 2 for primer sequences). RT-PCR was carried out at 95°C for an initial 3 min followed by 40 cycles of denaturation at 95°C for 10 s and annealing at 65°C for 45 s (ICAM1, KLF2), at 56°C for 45 s (VCAM1, TNF). The ΔΔCt method was used to calculate fold changes in expression of target RNAs (51): ΔCt = Ct (target gene)–Ct (housekeeping gene), ΔΔCt = ΔCt (treatment)–ΔCt (control), and fold change = 2^(−ΔΔ*Ct*)^.

**Table 2:** List of primers.

**Primers**	**Sequences**
GAPDH-Fwd	ggt ggt ctc ctc tga ctt caa
GAPDH-Rev	gtt gct gta gcc aaa ttc
VCAM1-Fwd	ccg gat tgc tgc tca gat tgg a
VCAM1-Rev	agc gtg gaa ttg gtc ccc tca
ICAM1-Fwd	gtc ccc tca aaa gtc atc c
ICAM1-Rev	Aac ccc att cag cgt cac c
TNF-Fwd	ccc agg gac ctc tct cta atc
TNF-Rev	atg ggc tac agg ctt gtc act
KLF2-Fwd	gca cgc aca cag gtg aga ag
KLF2-Rev	acc agt cac agt ttg gga ggg
KLF4-Fwd	acc agg cac tac cgt aaa cac a
KLF4-Rev	ggt ccg acc tgg aaa atg ct
eNOS-Fwd	gtg gct gtc tgc atg gac ct
eNOS-Rev	cca cga tgg tga ctt tgg ct
SENP2-Fwd	agc ctg gtg gtg att gac cta aga
SENP2-Rev	agc tgt tga ggga atc tcg tgt ggt

### ERK5 transcriptional activity assay (mammalian one-hybrid assay)

Sub-confluent ECs plated on 6-well-plate were incubated in Opti-MEM medium (Invitrogen, Carlsbad, CA, USA) containing Plus-Lipofectamine transfection reagents, the pG5 luciferase (pG5-Luc) and pBIND-ERK5 plasmids with pcDNA3.1-CA-MEK5α or control pcDNA3.1 vector, as we performed and described previously (41), for up to 4 h. Then, the transfection mixture was removed, ECs were washed, and ECM was added. Next, cells were treated with ponatinib at the concentrations indicated in the figures, for 24 h. Finally, cells were harvested, lysed, and luciferase activity was measured by a TD-20/20 Luminometer (Turner Designs, Sunnyvale, CA, USA), using dual-luciferase reporter reagents (Promega, Madison, WI, USA). The pG5-Luc plasmid has five Gal4 binding sites upstream of a minimal TATA box, which in turn, is upstream of the firefly luciferase gene. The pBIND-ERK5 plasmid has Gal4 fused with ERK5. Because pBIND vector also contains the Renilla luciferase gene, the expression and transfection efficiency were normalized to the Renilla luciferase activity. Therefore, relative ERK5 transcriptional activity was calculated by normalizing firefly luciferase activity according to Renilla luciferase activity (firefly:renilla luciferase activity ratio).

### Flow study

Confluent HAECs cultured in 100-mm dishes were exposed to laminar flow using a cone-and-plate apparatus placed in an incubator at 37°C and 5% CO_2_ for 24 h, as we previously described (52).

### KLF2 promoter activity

Sub-confluent HAECs were transfected with Flag-ERK5, a reporter gene encoding KLF2 promoter (−924 ~ + 14) and the luciferase control reporter vector pRL-CMV, using an OPTI-MEM/Plus-Lipofectamine mix, as we previously described (52). After incubating 3 h at 37°C, the transfection mix was removed and replaced with complete ECM. Next day, complete ECM was replaced with low serum ECM (0.2% FBS, 1% P/S, no ECGF). After 1 h, ponatinib (150 nM) was added to the medium and cells were incubated an additional 24 h. KLF2 promoter luciferase activity was assayed using a dual-luciferase reporter system.

### Immuno-precipitation (IP, to detect ERK5-SUMOylation), SDS/PAGE and immuno-blotting (IB)

At the end of experiments, ECs were washed three times in ice-cold PBS and lysed by adding a sufficient volume of 1X cell lysis buffer (cat. no. 9803S; Cell Signaling Technology, Danvers, MA, USA) or modified RIPA buffer (50 mM Tris-HCl, pH 7.4, 150 mM NaCl, 1 mM ethylenediaminetetraacetic acid, 1% Nonidet P-40, 0.1% sodium dodecyl sulfate (SDS), 0.25% sodium deoxycholate) supplemented with a mammalian protease inhibitor cocktail (cat. no. p8340; Sigma, St. Louis, MO, USA), 1 mM phenylmethylsulfonyl fluoride (cat. no. 36978; Thermo Fisher Scientific, Waltham, MA, USA), and 20 mM N-ethylmaleimide (cat. no. E3876; Sigma, St. Louis, MO, USA). The resulted cell lysates were centrifuged at 15,000 rpm for 15 min, and supernatants were collected. Protein concentrations were determined using a standard BCA protein assay. For IP, anti-ERK5 was added to the cell lysates followed by overnight incubation in cold room, with rocking. Next, a mixture of protein A/G agarose [1:1 ratio protein A agarose (cat. no. 15918-014; Invitrogen, Carlsbad, CA, USA) and recombinant protein G agarose (cat. no. 15920-010; Invitrogen, Carlsbad, CA, USA)] was added and incubated further. Beads were then washed three times with ice-cold lysis buffer, and bound proteins were released in 2X SDS sample buffer and analyzed by IB with anti-SUMO to detect SUMOylated ERK5. For IB, we loaded equal protein amounts from control and treated samples in each well of SDS-polyacrylamide gel, and proteins were resolved using SDS-polyacrylamide gel electrophoresis and electro-transferred onto Immobilon polyvinylidene fluoride transfer membranes (cat. no. IPVH00010; EMD Millipore, Darmstadt, Germany). The membranes were then immunoblotted with an antibody against each indicated protein. We incubated with the primary antibodies at 1:1,000 and at 1:5,000 dilutions for goat anti-mouse or anti-rabbit secondary antibodies conjugated with HRP. Resulted membranes were visualized using an enhanced chemiluminescence detection reagent (cat. no. 170-5060; Bio-Rad, Hercules, CA, USA) following the manufacturer's instructions.

### Automated capillary electrophoresis western analysis

Whole cell lysates were collected in modified RIPA buffer as described in the IP and IB section. A total of 5 μL of 0.4–1 mg/mL protein was loaded into plates and capillary electrophoresis western analysis was carried out following the manufacturer's instructions (Protein simple WES, part no. 004-600, ProteinSimple, San Jose, CA) using the 12–230 kDa Separation Module (part no. SM-W003, ProteinSimple, San Jose, CA) and either Rabbit (part no. DM-001, ProteinSimple, San Jose, CA) or Mouse (part no. DM-002, ProteinSimple, San Jose, CA) Detection Modules. Briefly, whole cell lysates were mixed with 5X fluorescent master mix containing 200 mM DTT followed by heating at 95°C for 5 min. Cell lysates, blocking buffer (antibody diluent), primary antibodies (in antibody diluent), HRP-conjugated secondary antibodies, and luminol-peroxide were then dispensed onto the separation plate. Antibodies against β-actin served as loading controls and were multiplexed with the primary antibodies for all samples. Capillary electrophoresis was performed using the instrument default settings: separation time 25 min, separation voltage 375 V, blocking 5 min, primary and secondary antibodies 30 min. Finally, automatically detected standards and peaks were manually inspected, and the data were analyzed with the inbuilt Compass software (ProteinSimple) (62).

### Assessment of barrier function by transepithelial/transendothelial electrical resistance (TEER) measurements

TEER values of HAEC monolayers treated with ponatinib was assessed in real-time by ECIS system using 8W10E+ array chambers. Briefly, the array chambers were treated with 10 mM L-Cysteine solution (room temperature, 15 min) followed by washing twice with ultra-pure water. The treated chambers were then coated with 0.2% gelatin type A. HAECs were seeded into the chambers and grown in complete ECM overnight to produce a confluent monolayer. Next day, complete ECM was replaced with low serum ECM (0.2% FBS, 1% P/S, no ECGF) and baseline resistance measurements were taken. Upon stabilization, ponatinib was added, and change in TEER values were recorded by an ECIS-Zθ instrument (Applied BioPhysics Inc., Troy, NY, USA) connected with a Dell personal computer equipped with ECIS software (Applied Biophysics). Figures illustrate normalized TERR values (where the value of 1.0 represents the basal TEER measurement immediately before adding ponatinib). Decrease in TEER indicates increased permeability (63).

### Flow cytometric analysis of apoptotic cells by annexin V staining

Following treatment (as indicated in the figures), cells were washed twice with PBS, harvested passively using 10 mM Ethylenediaminetetraacetic acid (EDTA, pH 8.0) solution at room temperature, and stained for apoptotic marker Annexin-V using Annexin V-FITC Apoptosis Detection Reagent (cat. no. ab14082; Abcam; Cambridge, MA, USA) as per the manufacturer's instructions. Briefly, cell pellets were re-suspended in 1X Annexin V Binding Buffer (cat. no. ab14084; Abcam; Cambridge, MA, USA) and baseline measurements were taken (unstained controls). Then, cells were stained with Annexin V-FITC (cat. no. ab14083; Abcam; Cambridge, MA, USA) at room temperature for 5 min in the dark. Measurements for all samples were carried out using Accuri C6 flow cytometer (BD Biosciences, CA, USA). Ten thousand cells were acquired based on forward and side scatter characteristics. Results were analyzed using FlowJo software (version 10.5.0, FlowJo LLC, USA).

### “scratch” wound closure assay

Confluent HAECs transduced with Ad-ERK5-WT, Ad-ERK5-K6/22R or Ad-LacZ were wounded using a 1000 μL microtip. Complete ECM medium was replaced with low serum ECM, and cells were treated with ponatinib. 48 h later, cells were photographed and wound closure ability was assessed by comparing the wound size of ponatinib and veh-treated cells.

### Statistics

Differences between two independent groups were determined using the student's *t*-test (two-tailed). Differences between multiple groups were determined using one-way analysis of variance (ANOVA) followed by Bonferroni *post hoc* testing for multiple group comparison by GraphPad Prism (GraphPad Software, San Diego, CA, USA). *P* values < 0.01 were considered statistically significant and are indicated by two asterisks in the figures. *P*-value < 0.001 is indicated by three asterisks.

## Results

### Ponatinib triggers an inflammatory response in ECs

**Figure 1:**
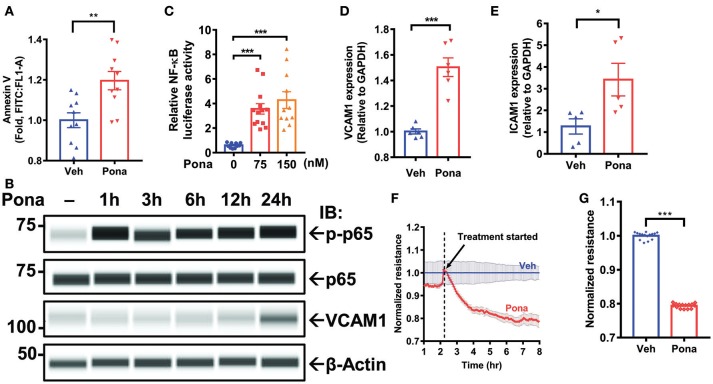
Ponatinib mediates endothelial inflammatory responses **(A)** Flow cytometric analysis of Annexin V staining in HAECs treated with ponatinib (150 nM, 24 h). Graph shows the fold increase of apoptosis in ponatinib treated cells compared to control cells. Data is sourced from two independent experiments, each contains 4–5 technical replicates. Error bars represent mean ± SEM. Statistical significance was assessed using student's *t*-test (two-tailed). ^**^p < 0.01. **(B)** Expression of phospho-p65, total p65, VCAM1, and β-actin (loading control) in HAECs treated with ponatinib was assessed by Protein simple WES system (capillary electrophoresis western analysis). Protein bands are shown as pseudoblots. **(C)** Graph demonstrates relative NF-kB activity, as measured by promoter-driven luciferase reporter gene assay in HAECs treated with phamacological concentrations of ponatinib for 24 h, and presented as ratio of firefly/renilla luciferase activity. A representative data set of three independent experiments is shown, contains 11–13 technical replicates. Error bars represent mean ± SEM. Statistical significance was assessed using ANOVA followed by Bonferroni *post hoc* testing for multiple group comparison. ^***^p < 0.001. **(D-E)** qRT-PCR analysis of relative *vcam1* and *icam1* expression in HAECs treated with ponatinib (150 nM, 24 h). A representative data set of three independent experiments is shown, contains 5–6 technical replicates. Error bars represent mean ± SEM. Statistical significance was assessed using student's *t*-test (two-tailed). ^***^P < 0.001; ^*^P < 0.05 vs. vehicle control (Veh) **(F)** Ponatinib (150 nM) effect on transcellular electrical resistance was assessed using ECIS system as described in methods. The dashed line indicates addition of ponatinib. Graph shows normalized resistance measured approximately every 4 min for 8 h, contains 4 technical replicates. Error bars represent mean ± SEM. **(G)** Graph demonstrates normalized resistance after 7 h of ponatinib treatment relative to Veh, contains 15 technical replicates. Error bars represent mean ± SEM. Statistical significance was assessed using student's *t*-test (two-tailed). ^***^P < 0.001.

Cell viability, migration, and functionality are decreased in HUVECs treated with ponatinib (35). To examine if the observed effect is similar across different types of ECs, we treated HAECs with ponatinib to assess apoptosis. Flow cytometric analysis of Annexin V staining indicated increased cell apoptosis in ponatinib treated group (Figure 1A). Because dying cells trigger an inflammatory response (64), we asked if ponatinib-associated apoptosis triggers an endothelial inflammatory response. In HAECs treated with pharmacologically relevant concentrations of ponatinib (75 nM, 150 nM) (35, 65), we noted a significant increase on NF-kB p65 phosphorylation (Figure 1B) and NF-κB activity (Figure 1C). Expression of inflammatory genes including vascular and intercellular cell adhesion molecule 1 (*vcam1* and *icam1*) was also increased, both at mRNA (Figures 1D,E) and protein levels (Figure 1B) in ponatinib treated cells. EC apoptosis can lead to disruption of the EC barrier that results in vascular leakage (64, 66). Using electric cell-substrate impedance sensing (ECIS) system, we assessed the effect of ponatinib on EC barrier function by measuring transendothelial electrical resistance (TEER) of cell monolayers. TEER values revealed increased EC permeability in ponatinib treated cells (Figures 1F,G). Taken together, our results suggest that ponatinib induces apoptosis along with and inflammatory response in ECs.

### Ponatinib inhibits ERK5 transcriptional activity

**Figure 2:**
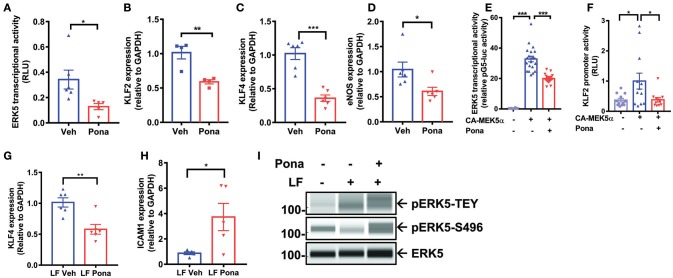
Ponatinib inhibits ERK5 transcriptional activity **(A)** Graph demonstrates relative ERK5 transcriptional activity, as measured by mammalian one-hybrid assay, in HAECs treated with ponatinib (150 nM, 24 h), and presented as ratio of firefly/renilla luciferase activity. A representative data set of three independent experiments is shown, contains 6 technical replicates. Error bars represent mean ± SEM. Statistical significance was assessed using student's *t*-test (two-tailed). ^*^P < 0.05 vs. Veh control. **(B-D)** qRT-PCR analysis of relative *klf2, klf4*, and *enos* expression in HAECs treated with ponatinib (150 nM, 24 h). A representative data set is shown, contains 4–6 technical replicates. Error bars represent mean ± SEM. Statistical significance was assessed using student's *t*-test (two-tailed). ^***^P < 0.001; ^**^P < 0.01; ^*^P < 0.05 vs. Veh control. **(E)** Graph demonstrate relative ERK5 transcriptional activity, as measured by mammalian one-hybrid assay in HAECs treated with ponatinib (150 nM, 24 h). Results are presented as ratio of firefly/renilla luciferase activity. As indicated, some cells were also transfected with CA-MEK5α. A representative data set of three independent experiments is shown, contains 11–20 technical replicates. Error bars represent mean ± SEM. Statistical significant was assessed using ANOVA followed by Bonferroni *post hoc* testing for multiple group comparison. ^***^P < 0.001 vs. Veh control. **(F)** Graph demonstrates relative KLF2 promoter activity, as measured by promoter-driven luciferase reporter gene assay in HAECs treated with ponatinib (150 nM, 24 h), and presented as ratio of firefly/renilla luciferase activity. As indicated, some cells were also transfected with CA-MEK5α. Error bars represent mean ± SEM. Data set contains 10–12 technical replicates. Statistical significance was assessed using student's *t*-test (two-tailed). ^*^P < 0.05 vs. Veh control. **(G-H)** qRT-PCR analysis of relative *klf4* and *icam1* expression in HAECs treated with ponatinib (150 nM, 24 h) in the presence of laminar flow. Data set contains 5–6 technical replicates. Error bars represent mean ± SEM. Statistical significance was assessed using student's *t*-test (two-tailed). ^*^P < 0.05; ^**^P < 0.01; vs. Veh control. **(I)** Expression of phospho-ERK5 at T218/Y220 (TEY), S496, and total ERK5 in HAECs treated with ponatinib (150 nM, 30 min) was assessed by Protein simple WES system (capillary electrophoresis western analysis). Protein bands are shown as pseudoblots.

Endothelial ERK5 plays a crucial role in vascular homeostasis, and its reduction leads to an accelerated inflammatory response in ECs (52). We asked if ponatinib triggers an endothelial inflammatory response via reducing ERK5 function. Employing a mammalian-one-hybrid assay, we noted decreased ERK5 transcriptional activity in ponatinib treated cells (Figure 2A). Similarly, expression of ERK5 responsive genes, including *klf2/4* and *eNOS*, was also inhibited (Figures 2B–D). In HAECs over-expressing a constitutive active form of MEK5α (CAMEK5α), ERK5 transcriptional activity (Figure 2E, bar 2 from the left) and KLF2 promoter activity (Figure 2F, bar 2 from the left) were activated, both of which were inhibited upon ponatinib treatment (Figures 2E,F, bar 3). Ponatinib also inhibited the increase of *klf4* (Figure 2G) and decrease of *icam1* expression (Figure 2H) induced by laminar flow (43). These results indicate that ponatinib reduces ERK5 transcriptional activity. Since ERK5 transcriptional activity is regulated by activation of its kinase domain, we studied the effect of ponatinib on ERK5 T218/Y220 phosphorylation (pERK5-TEY). We found that ponatinib did not affect laminar-flow induced ERK5 T218/Y220 phosphorylation (Figure 2I, first lane) suggesting that ponatinib-mediated reduced ERK5 transcriptional activity is independent of ERK5 kinase activity. Interestingly, we found that after ponatinib treatment, ERK5 S496 phosphorylation was significantly increased, even in the presence of protective laminar flow (Figure 2I, second lane). Because ERK5 S496 phosphorylation promotes an inflammatory response in EC (52), ponatinib-mediated phosphorylation at this residue might play a crucial role in ponatinib-associated endothelial inflammatory response.

### Ponatinib increases ERK5 SUMOylation in ECs

**Figure 3:**
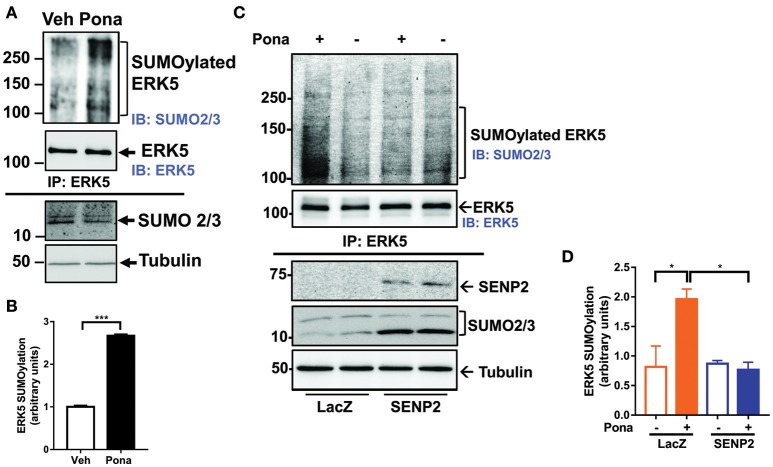
Ponatinib increases ERK5 SUMOylation. **(A,B)** Western blotting analysis of immuno-precipitated samples to detect SUMOylated ERK5 and ERK5 in HUVECs treated with ponatinib (150 nM, 60 min). A representative data set of two independent experiments is shown **(A)**. Densitometric quantification of SUMOylated ERK5. Data is sourced from two independent experiments, each contains 2 technical replicates. Error bars represent mean ± SEM. Statistical significance was assessed using student's *t*-test (two-tailed). ^***^p < 0.001 **(B)**. **(C,D)** Western blotting analysis of immuno-precipitated samples to detect SUMOylated ERK5 and ERK5 in HAECs, transduced with Ad-LacZ or Ad-SENP2, treated with ponatinib (150 nM, 60 min). A representative data set of three independent experiments is shown **(C)**. Densitometric quantification of SUMOylated ERK5. Data is sourced from three independent experiments, each contains 2 technical replicates. Error bars represent mean ± SEM. Statistical significance was assessed using ANOVA followed by Bonferroni *post hoc* testing for multiple group comparison. ^*^P < 0.05 vs. control.

ERK5 SUMOylation regulates endothelial inflammatory response via repressing laminar flow-mediated ERK5 transcriptional activation (60). We tested if ponatinib inhibits ERK5 transcriptional activity by promoting ERK5 SUMOylation. Following ponatinib treatment, we collected cell lysates for IP studies to determine the level SUMOylated ERK5 in ECs. We found that ERK5 SUMOylation was significantly increased in both HUVECs (Figure 3A,B) and HAECs (Figures 3C,D) treated with ponatinib, and that this increase was reversed in cells overexpressing deSUMOylation enzyme Sentrin/SUMO-specific protease 2 (SENP2) (Figures 3C,D).

### Ponatinib elicits endothelial inflammatory responses via promoting ERK5 SUMOylation

**Figure 4:**
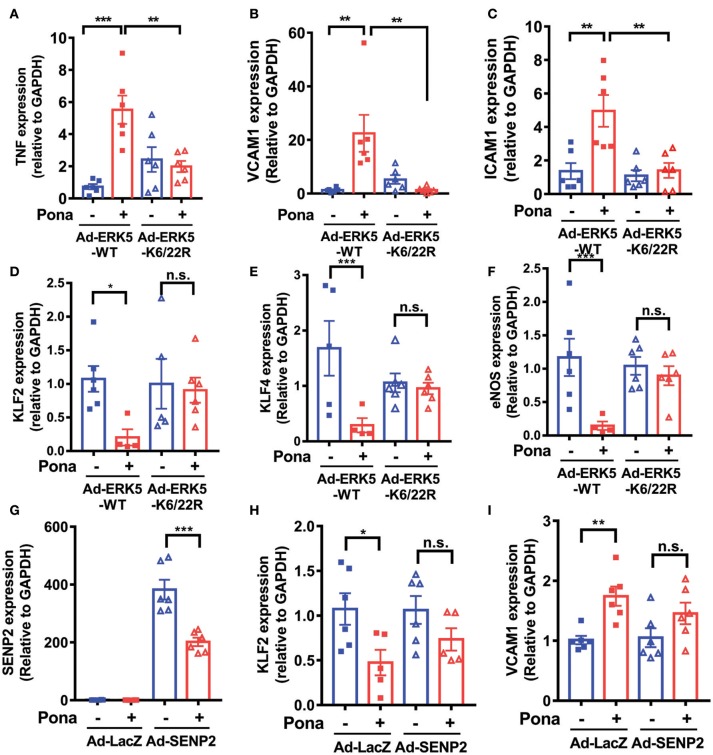
Ponatinib elicits endothelial inflammatory responses via promoting ERK5 SUMOylation. qRT-PCR analysis of relative *tnf, vcam1, icam1, klf2, klf4, eNOS*, and *senp2* in HAECs expressing ERK5-WT or K6/22R mutant **(A–F)**, and SENP2 **(G–I)** treated with ponatinib (150 nM, 24 h). Data set contains 5–6 technical replicates. Error bars represent mean ± SEM. Statistical significance was assessed using ANOVA followed by Bonferroni *post hoc* testing for multiple group comparison. ^***^P < 0.001; ^**^P < 0.01; ^*^P < 0.05 vs. control.

To verify the involvement of ERK5 SUMOylation in ponatinib-associated endothelial inflammatory response, we transduced HAECs with either an adenovirus expressing ERK5 wild type (Ad-ERK5-WT) or ERK5 non-SUMOylatable mutant (Ad-K6/K22R). The cells were then treated with ponatinib for 24 h, and expression of various genes were determined. qRT-PCR analysis revealed that the increased *tnf*α, *vcam1*, and *icam-1* as well as the decreased *klf2/4* and *enos* expression by ponatinib seen in ECs expressing ERK5-WT was reversed in ECs expressing the ERK5 K6/22R mutant (Figure 4A–F). It is possible that ERK5-WT over-expression might skew the involvement of ERK5 SUMOylation in ponatinib-mediated endothelial inflammatory response. Thus, in an independent experiment, we used SENP2 overexpression to inhibit endogenous ERK5 SUMOylation (Figures 3C, 4G), and found that the reduced ERK5 SUMOylation by SENP2 could partially reverse ponatinib's effect on *klf2* and *vcam1* expression (Figures 4H,I).

**Figure 5:**
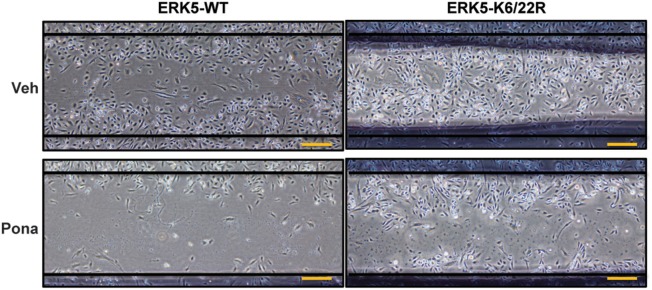
Delayed migration of cells toward the wounded region seen in ECs expressing ERK5-WT was rescued in ECs expressing the ERK5 K6/22R mutant: Confluent monolayers of HAECs expressing Ad-ERK5-WT or Ad-ERK5-K6/22R mutant were serum starved for 1 h, and then wounded with a 1000 μL pipette tip followed by ponatinib treatment (150 nM, 48 h). Cells were photographed and assessed for wound closure. Scale bar represent 100 μM. A representative data set of two independent experiments is shown.

A functional characteristic of ECs is their ability to migrate. During physiological processes, EC migrate during vasculogenesis and angiogenesis whereas in pathological process, such as vessel damage, EC migrate to restore vessel integrity (67). To investigate the role of ponatinib-mediated ERK5 SUMOylation in EC function, we performed an *in vitro* scratch wound healing assay. Of note, we minimized the contribution of cell proliferation in this process by: (1) wounding a confluent and quiescent monolayer of cells and (2) maintaining the cells in reduced-serum culture medium for the duration of the assay. In ECs expressing ERK5-WT, cell migration toward the wounded region seen in the veh-treated group was inhibited by ponatinib treatment (Figure 5, left panel). However, in ECs expressing ERK5-K6/22R mutant, ponatinib-mediated delayed cell migration was rescued (Figure 5, right panel). Taken together, our data suggests the importance of endothelial ERK5 SUMOylation in ponatinib-associated endothelial inflammatory response, and, to a greater extent, vascular adverse events.

## Discussion

Previously, we reported that p90RSK activation increases SENP2 T368 phosphorylation that inhibits SENP2 deSUMOylation activity, leading to increased ERK5 SUMOylation. Additionally, p90RSK activation increases ERK5 S496 phosphorylation. Both, SUMOylation and S496 phosphorylation reduce ERK5 transcriptional activity that accelerates EC inflammation, dysfunction, apoptosis, and subsequent atherosclerotic plaque formation (52, 61). In the current study, we identified a novel role for ERK5 SUMOylation in ponatinib-mediated endothelial inflammatory response. Interestingly, we also detected increased p90RSK phosphorylation by ponatinib (Supplementary Figure 1A). This signaling pathway elicited by ponatinib resembles that of ECs exposed to atheroprone stimuli, such as disturbed flow, reactive oxygen species, or advanced glycation end products (52, 61), suggesting that ponatinib-mediated ERK5 SUMOylation might be involved in ponatinib-associated atherosclerosis, VAEs and subsequent cardiovascular complications. Thus, inhibition of endothelial ERK5 SUMOylation can be viewed as a novel approach to mitigate VAEs resulting from ponatinib treatment.

Because we also found decreased SENP2 expression in ECs expressing SENP2 treated with ponatinib (Figure 4G), we speculate that ponatinib elicits an endothelial inflammatory response not only by reducing SENP2 activity but also by reducing SENP2 at the protein expression level. This might be a unique feature of ponatinib, compared to other pro-inflammatory stimuli where effects on SENP2 are only on its enzymatic activity.

Endothelial ERK5 can be phosphorylated at multiple sites, each of which confers different biological functions (51, 52, 68–70). Among them, ERK5 S496 phosphorylation plays a crucial role in EC inflammation. ERK5 S496 phosphorylation has a similar effect to that of ERK5 SUMOylation in inhibiting ERK5 transcriptional activity (52). We reported previously that ERK5 phosphorylation at S496 is induced not only by disturbed flow and reactive oxygen species (52, 61) but also by radiation (IR) and that it plays a crucial role in IR-mediated EC inflammation (69). In the current study, we found that ERK5 S496 phosphorylation is increased by ponatinib (Figure 2I and Supplementary Figure 1A). Furthermore, ponatinib-mediated increased *tnf*α expression was reversed in ECs expressing ERK5 S496A phosphorylation resistant mutant (Supplementary Figure 1B). Similarly, flow cytometric analysis of Annexin V staining revealed the contribution of ERK5 S496 phosphorylation in ponatinib-induced EC apoptosis (Supplementary Figure 1C).

It is noteworthy that the reduction of ERK5 transcriptional activity by ponatinib via ERK5 SUMOylation and, probably, S496 phosphorylation occurs independently of kinase activation, highlighting the predominance of these posttranslational modifications on ERK5 function and, subsequently, EC integrity. If and how ponatinib-induced ERK5 SUMOylation and S496 phosphorylation interact and/or coordinate to control ERK5 transcriptional activity requires further investigation. To the best of our knowledge, this is the first study to demonstrate the role of endothelial ERK5 SUMOylation in ponatinib-regulated EC inflammation.

## Author contributions

JP-M, AC, RA, EH, YT, and N-TL performed experiments, interpreted data. JP-M, SK, BD, M-CH, and N-TL wrote and edited the manuscript. All authors have read and agreed to the content of the manuscript.

### Conflict of interest statement

The authors declare that the research was conducted in the absence of any commercial or financial relationships that could be construed as a potential conflict of interest.

## Supplementary material

Supplementary Figure 1 Ponatinib-mediated ERK5-S496 phosphorylation in endothelial inflammatory responses. **(A)** Expression of phospho-ERK5 at S496, ERK5, phospho-p90RSK at S380, and p90RSK in HAECs treated with ponatinib (150 nM) was assessed by Protein simple WES system (capillary electrophoresis western analysis). Protein bands are shown as pseudoblots. **(B)** qRT-PCR analysis of relative *tnf* expression in HAECs expressing ERK5-WT or S496A mutant treated with ponatinib (75 nM, 24 h). Data set contains 6 technical replicates. Error bars represent mean ± SEM. Statistical significance was assessed using ANOVA followed by Bonferroni *post hoc* testing for multiple group comparison. ^***^*P* < 0.001 vs. control. **(C)** Flow cytometric analysis of Annexin V staining in HAECs expressing ERK5-WT or S496A mutant treated with ponatinib (150 nM, 24 h). Graph shows percentage of apoptotic cells. Data set contains 3–6 technical replicates. Error bars represent mean ± SEM. Statistical significance was assessed using student's *t*-test (two-tailed). ^***^*p* < 0.001.

## Abbreviations

VAEs: Vascular adverse events
TKI: Tyrosine kinase inhibitor
Pona: Ponatinib
CML: Chronic myeloid leukemia
ERK5: Extracellular signal regulated kinase 5
VCAM1: Vascular cell adhesion molecule 1
ICAM1: Intercellular adhesion molecule 1
TNF: Tumor necrosis factor
SUMO: Small ubiquitin-like modifier
ECs: Endothelial cells
Ad: Adenovirus
HUVECs: Human umbilical vein ECs
HAECs: Human Aortic ECs
CAMEK5α: Constitutive active form of MEK5α
LF: Laminar flow
p90RSK: 90 kDa ribosomal protein S6 kinase
SENP2: Sentrin/SUMO-specific protease 2
KLF2/4: Krüppel-like Factor 2/4
eNOS: Endothelial nitric oxide synthase.
