# Supplementary figures and images for: Ponatinib Activates an Inflammatory Response in Endothelial Cells via ERK5 SUMOylation
Source: Front Cardiovasc Med. 2018 Sep 6;5:125. doi: 10.3389/fcvm.2018.00125 (PMC6135907; doi:10.3389/fcvm.2018.00125)

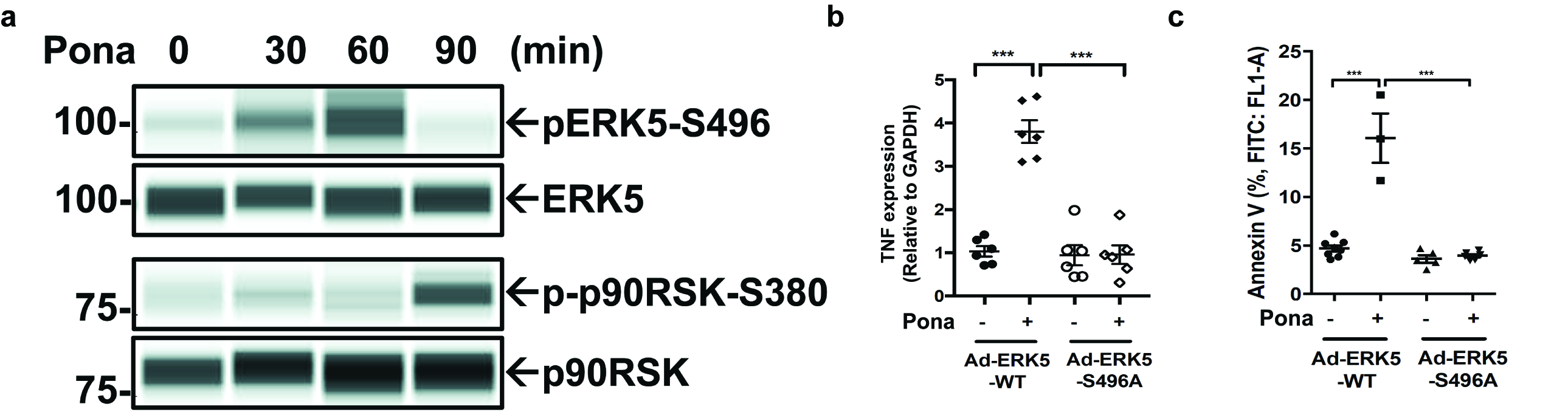

Supplement: Supplementary Figure 1 — Ponatinib-mediated ERK5-S496 phosphorylation in endothelial inflammatory responses. (A) Expression of phospho-ERK5 at S496, ERK5, phospho-p90RSK at S380, and p90RSK in HAECs treated with ponatinib (150 nM) was assessed by Protein simple WES system (capillary electrophoresis western analysis). Protein bands are shown as pseudoblots. (B) qRT-PCR analysis of relative tnf expression in HAECs expressing ERK5-WT or S496A mutant treated with ponatinib (75 nM, 24 h). Data set contains 6 technical replicates. Error bars represent mean ± SEM. Statistical significance was assessed using ANOVA followed by Bonferroni post hoc testing for multiple group comparison. ***P < 0.001 vs. control. (C) Flow cytometric analysis of Annexin V staining in HAECs expressing ERK5-WT or S496A mutant treated with ponatinib (150 nM, 24 h). Graph shows percentage of apoptotic cells. Data set contains 3–6 technical replicates. Error bars represent mean ± SEM. Statistical significance was assessed using student's t-test (two-tailed). ***p < 0.001. [file Image_1.TIF]
